# Marqibo^®^ (vincristine sulfate liposome injection) improves the pharmacokinetics and pharmacodynamics of vincristine

**DOI:** 10.1007/s00280-012-2042-4

**Published:** 2012-12-05

**Authors:** Jeffrey A. Silverman, Steven R. Deitcher

**Affiliations:** Talon Therapeutics Inc., 400 Oyster Point Blvd, Suite 200, South San Francisco, CA 94080 USA

**Keywords:** Liposome, Marqibo, Pharmacokinetics, Vincristine, VSLI, Xenograft

## Abstract

Vincristine (VCR) is a mainstay of treatment of hematologic malignancies and solid tumors due to its well-defined mechanism of action, demonstrated anticancer activity and its ability to be combined with other agents. VCR is an M-phase cell cycle-specific anticancer drug with activity that is concentration and exposure duration dependent. The pharmacokinetic profile of standard VCR is described by a bi-exponential elimination pattern with a very fast initial distribution half-life followed by a longer elimination half-life. VCR also has a large volume of distribution, suggesting diffuse distribution and tissue binding. These properties may limit optimal drug exposure and delivery to target tissues as well as clinical utility as a single agent or as an effective component of multi-agent regimens. Vincristine sulfate liposome injection (VSLI), Marqibo^®^, is a sphingomyelin and cholesterol-based nanoparticle formulation of VCR that was designed to overcome the dosing and pharmacokinetic limitations of standard VCR. VSLI was developed to increase the circulation time, optimize delivery to target tissues and facilitate dose intensification without increasing toxicity. In xenograft studies in mice, VSLI had a higher maximum tolerated dose, superior antitumor activity and delivered higher amounts of active drug to target tissues compared to standard VCR. VSLI recently received accelerated FDA approval for use in adults with advanced, relapsed and refractory Philadelphia chromosome-negative ALL and is in development for untreated adult ALL, pediatric ALL and untreated aggressive NHL. Here, we summarize the nonclinical data for VSLI that support its continued clinical development and recent approval for use in adult ALL.

## Introduction

Introduced over 45 years ago, vincristine (VCR) remains a potent and widely used anticancer agent, particularly for childhood and adult hematologic malignancies and solid tumors including sarcomas. However, sub-optimal pharmacokinetic properties and dose-related neurotoxicity prevent realization of the full potential of this agent. VSLI (vincristine sulfate liposome injection, 0.16 mg/mL (Marqibo^®^)) is a novel formulation of VCR that encapsulates the drug in sphingomyelin and cholesterol nanoparticles. The VSLI liposome is distinct from alternate liposomes used in other approved pharmaceutical products and is uniquely suited to contain, deliver, and dose intensify VCR. Here, we review the nonclinical investigations which demonstrate VSLI’s optimized pharmacokinetic profile, enhanced drug delivery to target cancer tissues and increased activity in tumor models. These and other nonclinical studies supported the clinical development of VSLI which led to the recent approval of VSLI by the US FDA for treatment of adult patients with Philadelphia chromosome-negative (Ph-) acute lymphoblastic leukemia (ALL) in second or greater relapse or whose disease has progressed following two or more antileukemia therapies.

## Vincristine background

VCR was initially discovered in a screening program investigating the potential antidiabetic properties of extracts from the widely cultivated white- or pink-flowered periwinkle plant, Cantharanthus roseus (formerly known as Vinca rosea Linn) [1–3]. Although ineffective as an oral antidiabetic treatment, the periwinkle extract was found to potently inhibit leukocyte production and maturation. Significant antileukemia activity of the alkaline fractions in animal models led to the subsequent isolation of several alkaloids, most potently from the leaves, which include the compounds now known as vincristine, vinblastine, vinleurosine and vinrosidine.

VCR is a highly active cell cycle-dependent anticancer drug. Extensive research on the mechanism of action of VCR has demonstrated that it binds to tubulin causing microtubule depolymerization, metaphase arrest and apoptotic death of cells undergoing mitosis [4, 5]. Tubulin is essential for the normal polymerization of mitotic spindle microtubules. VCR binding to spindle microtubules alters spindle structure and function in a concentration-dependent manner. At low concentrations, VCR stabilizes the spindle apparatus which prevents chromosome segregation and results in metaphase arrest and inhibition of mitosis. At higher concentrations, disruption and total depolymerization of microtubules has been observed. The effect of short-term VCR exposure on mitotic arrest is reversible and cells can proceed normally through the cell cycle if the drug is removed. In contrast, long-term exposure to high concentrations of VCR results in lethal cytotoxicity [6–10]. Thus, the antitumor potency of VCR is dependent on the concentration and duration of exposure and the number of cells transiting through mitosis during the period of drug exposure. Interference of microtubule function also disrupts other cellular processes that involve microtubules, such as intracellular transport and cellular organization [4, 11, 12]. As a result of its interruption of microtubule function, especially evident during M-phase, cells accumulate in metaphase contributing to VCR-induced cytotoxicity [11, 13].

VCR-mediated antitumor activity may also include antivascular and antiangiogenic properties. In vitro, VCR inhibits the secretion of angiogenic factors such as vasculature endothelial growth factor (VEGF) by normal and drug-resistant human tumor cells [14], inhibits the proliferative activity and formation of capillary networks in cultured endothelial cell assays, and reduces the migratory activity of tumor cells in Matrigel assays [15]. In mouse models, VCR and other vinca alkaloids decrease vascular flow in tumors and normal tissues [16–19]. Antiangiogenic activity and decreased microvasculature density in tumor xenograft models have been described following VCR therapy [20]. The role of microtubules in these effects has not been established.

After clinical trials demonstrated anticancer activity in humans, the US FDA granted marketing approval for VCR in 1963. It has subsequently become an essential component of multi-drug chemotherapeutic regimens for the treatment of hematologic malignancies [21]. Early demonstration of VCR’s activity and dose–response relationship in acute leukemia and Hodgkin’s lymphoma led to clinical investigations in additional cancers [1]. Standard VCR has subsequently been approved for use in many neoplasms, including ALL, and in combination with other agents for the treatment of Hodgkin’s disease, non-Hodgkin’s lymphoma (NHL), rhabdomyosarcoma, neuroblastoma and Wilms’ tumor. The usual dose of VCR for adults is 1.4 mg/m^2^ administered intravenously once a week. However, the oncology community and most vincristine-containing cancer treatment regimens routinely limit individual standard vincristine doses to 2.0 mg (i.e., dose capping) in an attempt to minimize neurotoxicity.

**Fig. 1:**
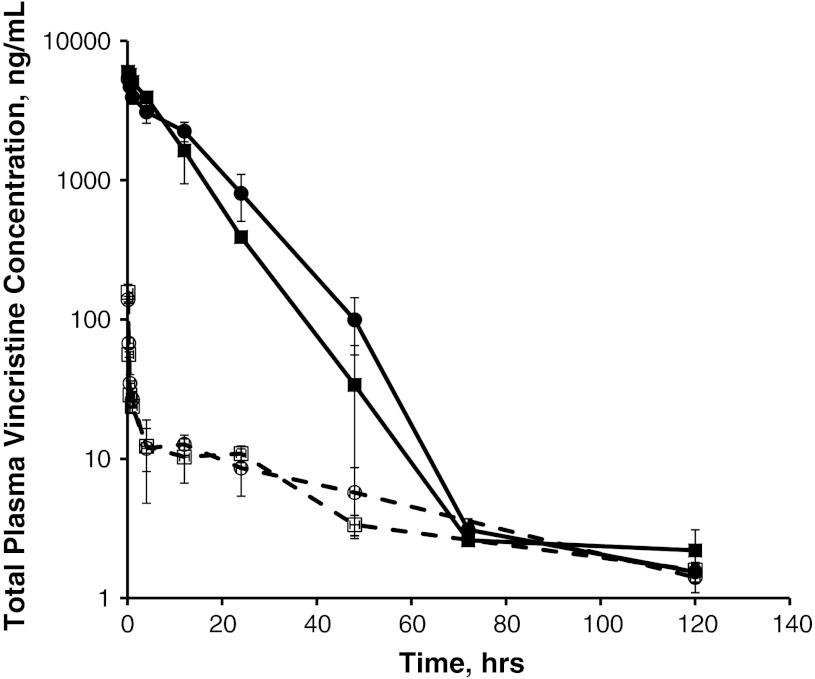
Plasma vincristine concentration following administration of 2 mg/m^2^ of VCR (*dashed lines*) or VSLI (*solid lines*) to rats. Vincristine drug concentrations were measured in plasma from Sprague–Dawley rats (*N* = 3/sex, except at 120 h, *N* = 5) at the indicated timepoints post-dose of 2 mg/m^2^ VSLI (*solid lines*) or vincristine (*dashed lines*). Symbols indicate the following: Male VCR (*open circle*), Female VCR (*open square*), Male VSLI (*filled circle*), Female VSLI (*filled square*)

Despite its potent antineoplastic activity, however, VCR has several pharmacological limitations. VCR plasma pharmacokinetics are described by a bi-exponential profile with a very short and extensive distribution half-life followed by a longer elimination half-life (Fig. 1); the volume of distribution is large, suggesting wide and diffuse distribution and perhaps extensive tissue binding (Table 1). These pharmacological properties may limit its optimal clinical benefit by limiting plasma and cancer tissue C_max_ and cancer tissue drug exposure.

**Table 1:** Pharmacokinetic parameters in VSLI- and VCR-treated rats at 2.0 mg/m^2^

Formulation	*C*_max_ (ng/mL)	AUC_inf_ (ng·h/mL)	*t*_1/2λ1_ (h)	*t*_1/2λz_ (h)	Cl (mL/h/m^2^)	*V*_ss_ (mL/m^2^)
VSLI	5,662	63,438	6.9	NA^a^	32	383
VCR	148	806	0.2	36.5	2,488	113,513

## Liposome overview

Liposomes are small phospholipid vesicles that are versatile drug carriers which can be used to overcome the potential barriers of many drugs and allow effective delivery to their target tissues such as tumors (reviewed in [22–27]). Liposomes are simple, self-assembling vesicles with either single phospholipid bilayers (unilamellar) or multiple phospholipid bilayers that enclose an aqueous core, which can include a therapeutic drug “payload”. Liposomes can be used to solve sub-optimal pharmaceutical properties such as low solubility, instability and rapid metabolism; they can also alter the distribution of drugs and offer the potential of selective delivery to the site of action [22, 28]. To be effective as a delivery system, liposomes balance stability and time in the systemic circulation with release, or bioavailability, of the drug at the target site. VCR exhibits low solubility in aqueous solutions at physiological pH in vitro and has a rapid initial plasma Cl and extensive volume of distribution in vivo (Fig. 1; Table 1) [4, 5, 12, 29]. These physico-chemical and pharmacokinetic properties combined with VCR’s narrow therapeutic index and strong anticancer activity make it well suited for liposome technology to improve its utility in cancer therapy.

For many liposome technology–enhanced drugs, particularly those that are slowly released from their liposome, the pharmacokinetic properties become similar to that of the liposome itself [25]. The initial formulation of liposomal VCR used distearoylphosphatidylcholine and cholesterol liposomes and a pH gradient to load the drug into the vesicles. This formulation demonstrated a longer circulation time, enhanced tumor delivery and antitumor activity and decreased toxicity compared to the standard formulation of VCR [29–31]. Subsequent development of liposomal formulations for VCR to optimize its pharmacokinetic properties, such as increased circulation time and enhanced delivery of the drug to target tissues, led to the identification and development of sphingomyelin/cholesterol (SM/Chol) liposomes [32–34]. These SM/Chol liposomes offer the advantage of improved drug loading, retention and release, longer plasma circulation time and enhanced target-tissue accumulation without the technical challenges, and manufacturing expense, of surface-modified liposome technologies, for example, liposomes using Polyethylene Glycol (PEG) polymers.

## VSLI overview

VSLI, Marqibo^®^, is a proprietary sphingomyelin- and cholesterol-based nanoparticle formulation of VCR that was designed to overcome the dosing and pharmacokinetic limitations of standard VCR. As described above, prolonged exposure of cells to VCR enhances its in vitro cytotoxicity due to the fact that at longer exposure times a greater proportion of the cells will have passed through mitosis, where VCR exerts its cytotoxic effects [35–37]. The liposomal carrier component of VSLI, composed of sphingomyelin and cholesterol, was specifically designed to facilitate the loading and retention of VCR, to prolong the circulation time of encapsulated VCR, to increase extravasation into tumors and to slowly release the drug in the tumor interstitium [38, 39]. These characteristics result in high levels of encapsulated drug in target tissues and a long duration of exposure of tumor cells to therapeutic drug concentrations as VCR is slowly released from the liposomes, leading to enhanced activity.

## VSLI nonclinical pharmacokinetics

VSLI has a long circulation time and remains in the plasma instead of being rapidly and widely distributed in tissues like unencapsulated VCR [40–42]. The clearance of liposomes is largely a function of uptake by the mononuclear phagocytic system (MPS) which is influenced by the lipid composition, size of the nanoparticle and the extent of protein binding, or opsonization, by serum proteins [25]. The SM/Chol lipid composition and the ~100 nm mean particle size of the VSLI liposome contribute to low protein binding that result in a longer circulation time for the nanoparticle [38, 43, 44]. In vitro protein binding assays demonstrated negligible levels (limit of detection 4.5 μg protein/mg lipid) of bovine or human plasma proteins adsorbed to VSLI which was consistent with the biophysical properties of the SM/Chol liposome, that is, uncharged and tight lipid packing [41, 44] (unpublished data, Talon Therapeutics). Approximately 18–39 % of encapsulated VCR was released at 24 h at 37 °C in an in vitro assay using human plasma. These characteristics of the liposome facilitate VSLI accumulation in tumors and tissues of the MPS due to the larger microvascular fenestrations in those tissues [40]. Subsequently, the nanoparticles slowly release the VCR in those tissues with an in vivo half-life of approximately 24 h.

The pharmacokinetic profile of VSLI was established in mice, rats and dogs. The pharmacokinetic properties of VSLI are consistent across species with VSLI showing substantially lower total VCR clearance and volume of distribution (V_ss_) and correspondingly greater area under the curve (AUC) than VCR (Table 2). These data show in all three species that, compared to VCR, VSLI is not rapidly distributed to tissues in the first few minutes after administration and that it remains in the systemic circulation and subsequently distributes into MPS tissues and tissues with fenestrated vasculature (e.g., bone marrow, lymph nodes, spleen and tumors). Linear relationships between VSLI dose and total VCR AUC and C_max_ (maximum concentration) were observed after single doses in rats over the dose range of 1.0–3.0 mg/m^2^ and in dogs over the range of 0.5–1.1 mg/m^2^ (not shown). Figure 1 and Table 1 illustrate the pharmacokinetic profile and calculated parameters of VSLI in rats following an IV dose. Following a brief initial decline, the reduction in total VCR concentration was minimal, suggesting a delay phase. The extensive early rapid distribution phase seen with VCR does not occur with VSLI. Total (encapsulated + free) VCR concentration declined monoexponentially. Notably, V_ss_ for total VCR after VSLI injection was close to plasma volume, indicating VSLI is confined within the plasma compartment for a longer period of time compared to VCR, and subsequently circulates repeatedly through target tissues and then accumulates in tissues with fenestrated vasculature, in particular, tumors and tissues of the MPS as described above. The disposition kinetics of the lipid component of VSLI was highly correlated with those for total VCR, indicating that the VCR is retained in the liposome and that the pharmacokinetics of VCR after VSLI administration is governed by the pharmacokinetics of the liposomes [40].

**Table 2:** Cross-species comparison of pharmacokinetic parameters

	Dose (mg/m^2^)	*C*_max_ (ng/mL)	AUC_inf_ (ng·h/mL)	*t*_1/2λ1_ (h)	*t*_1/2λz_ (h)	Cl (mL/h/m^2^)	*V*_ss_ (mL/m^2^)
Mouse^a^							
VCR	6	1,470	11,100	0.19	24.8	494	17,214
VSLI	6	22,600	351,100		10.8	16	242
Rat							
VCR	2	148	806	0.2	36.5	2,488	113,513
VSLI		6,271	89,910		6.0	23	244
Dog							
VCR	0.8	31	62	0.2	8.5	15,032	132,453
VSLI	0.8	656	543	0.2	10.6	1,474	5,436

^a^Determined from blood

**Fig. 2:**
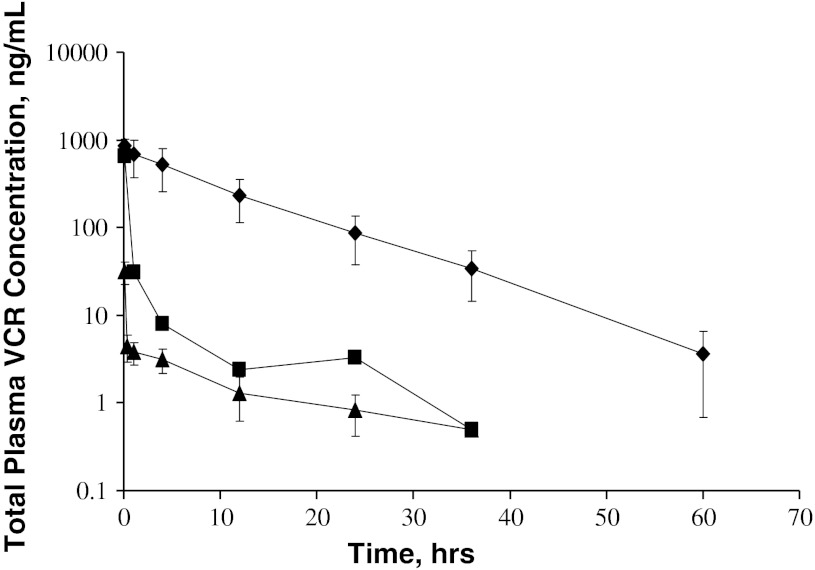
Plasma vincristine concentration following administration of 0.8 mg/m^2^ VCR or VSLI to dogs. Vincristine drug concentrations were measured in plasma from dogs at the indicated timepoints post-dose of VSLI or vincristine. Symbols indicate the following: VSLI Monoexponential (*N* = 12, *closed diamond*), VSLI Bi-exponential (*N* = 2, *filled square*), VCR (*N* = 14, *filled triangle*)

The pharmacokinetic profile in most dogs also showed an initial delay phase followed by a slow decline in total VCR levels with a prolonged half-life (Fig. 2). However, some dogs (17 %) showed an early rapid reduction followed by a slow decline in total plasma VCR that suggested a bi-exponential profile. Repeated doses of VSLI at 2 week intervals revealed that dogs retained their individual characteristics, that is, they continued to exhibit their mono or bi-exponential profile for the duration of the study. Analysis of the VCR in plasma demonstrated that >90 % of total VCR remained encapsulated so that the drug was not rapidly released in the systemic circulation. Thus, the difference in plasma pharmacokinetic profile was not due to release of the drug from the liposome and may represent differences in the capacity of individual animal MPS.

The extent of VCR metabolism and the metabolic profiles of VCR in rat urine and bile were similar for VSLI and VCR, indicating bioavailable VCR was metabolized similarly for both formulations. Radio-labeled mass-balance studies in rats showed that approximately 90 % of the administered dose of VSLI was recovered in the urine and feces over 72 h post-dose, a delay of 12–48 h compared to the standard VCR formulation. This is consistent with prolonged circulation of VSLI as well as the prolonged retention of VCR within the liposomes in vivo. From either formulation, less than 10–20 % of the total radioactivity in the bile and urine was present as metabolites (Talon Therapeutics, data on file and Castle et al. 1976, Castle and Mead 1978) [45, 46]. The major route of excretion of radioactivity in VSLI-treated rats was biliary since the majority of radioactivity (75 %) was recovered in the feces. The maximum fecal excretion occurred between 24 and 72 h, whereas the maximum urinary excretion occurred between 0 and 12 h. Combined, these data demonstrate retention of the encapsulated drug in the liposomes and slow release of the VCR in the target tissues.

**Fig. 3:**
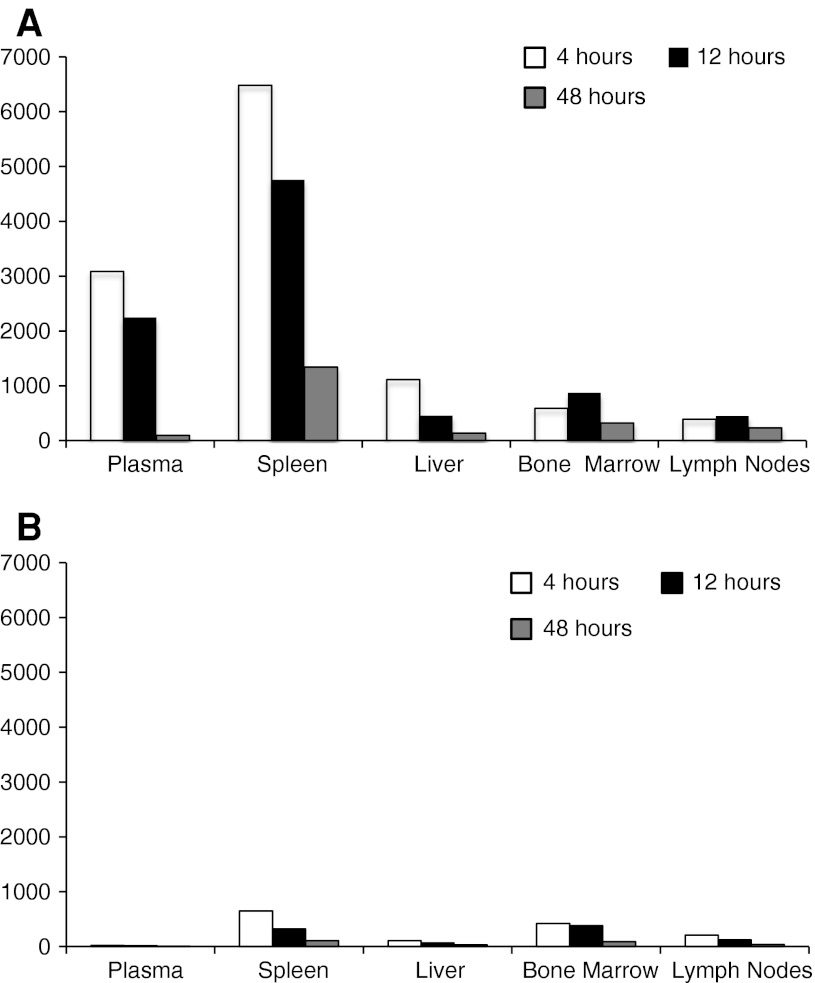
Tissue total vincristine concentration following administration of VSLI (**a**) or VCR (**b**) to rats. Rats were administered a single bolus dose of 2.0 mg/m^2^ of either [^3^H]Vincristine or VSLI that had [^3^H]Vincristine encapsulated into the liposome. Tissue drug levels were measured up to 72 h post-dose by liquid scintillation counting of tissue samples. The *bars* represent samples taken at the following times: 4 h (*open bars*), 12 h (*black bars*), 24 h (*gray bars*)

The comparative tissue distribution of total VCR following a single IV bolus injection of either VSLI or VCR was assessed in rats and mice. Following administration of VSLI, the maximum plasma concentration (*C*_max_) of VCR in most tissues was between 4 and 24 h with the majority of tissues peaking around 16 h. For most tissues from VSLI-treated animals, tissue to plasma concentration ratios increased over time and peaked at 72 h after VSLI injection, indicating progressive accumulation of radio-labeled drug from plasma into the tissues [42, 47]. The tissue distribution of total VCR was slower following administration of VSLI than after VCR, and more extensive accumulation was observed in tissues of the MPS such as the spleen, liver, lymph nodes and bone marrow (Fig. 3). The rank order of tissues based on *C*_max_ demonstrated that total VCR concentrations in MPS tissues and in ovaries were substantially higher than in other organs or tissues. The lowest radioactivity levels were observed in brain, spinal cord, nerves and muscle. Higher exposures, as measured by AUC_inf_, of VCR were observed in spleen (12 fold), lymph nodes (tenfold), liver (fourfold) and bone marrow (twofold) following a radio-labeled dose of VSLI compared to VCR (Table 3). Thus, preferential accumulation of the liposomes and subsequent slow release of VCR in target tissues important in hematologic malignancies following administration of VSLI results in higher and prolonged tissue drug levels providing superior drug delivery to those tissues than the same dose of standard formulation of VCR.

**Table 3:** Comparison of tissue exposure to vincristine following administration of either VSLI or VCR

	AUC_inf_, ng eq· h/g			
	Spleen	Liver	Lymph node	Bone marrow
VSLI	381,154	28,202	66,333	40,936
VCR	30,765	6,496	6,420	17,767
Ratio VSLI/VCR	12.4	4.3	10.3	2.3

The extravasation kinetics and preferential accumulation of VSLI in tumors was further investigated using intra-vital microscopy imaging in mice implanted with LX-1 cells, a xenograft model of human small-cell carcinoma [48]. Significantly faster extravasation occurred in tumor vessels than in nontumor tissues after a single dose of fluorescently labeled VSLI liposomes. The interstitial amounts of drug were approximately 70-fold higher in tumor tissues compared to nontumor tissues at 1 h and remained higher at 48 h. Combined, these distribution data are consistent with VSLI exiting the systemic circulation, accumulating at the site of tumors where they act as a reservoir for the release of localized VCR to enhance the antitumor activity.

## VSLI nonclinical pharmacodynamics

**Fig. 4:**
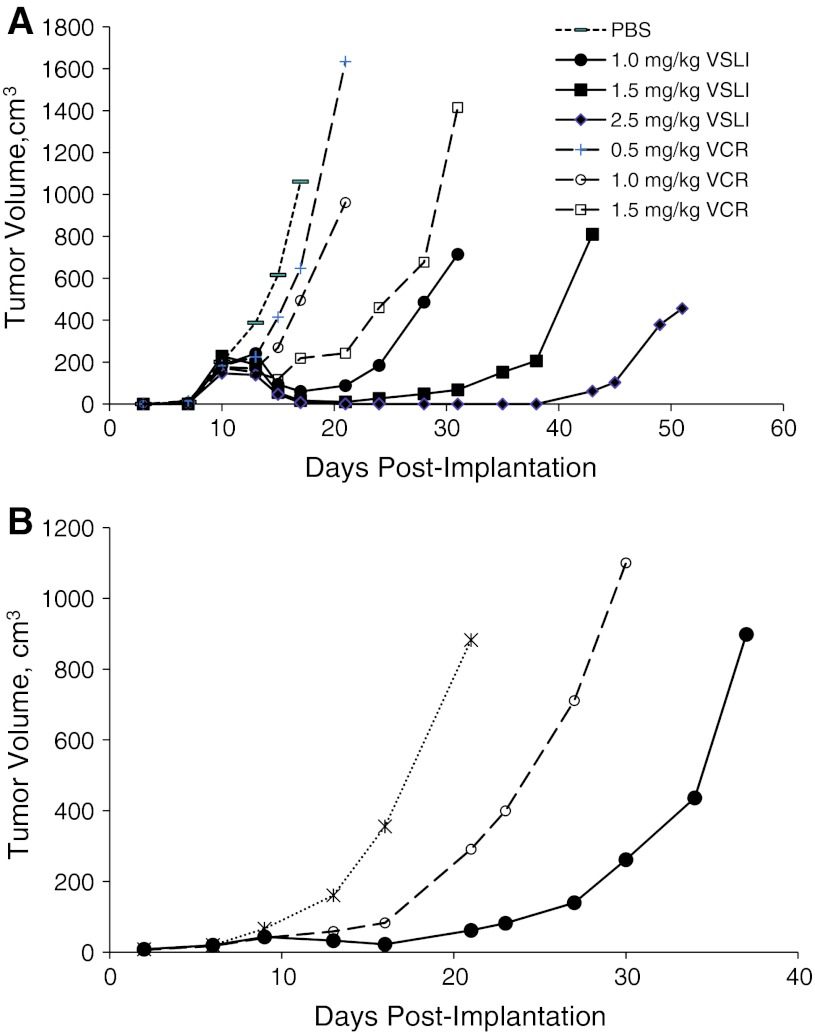
VSLI and VCR antitumor activity in Namawala (**a**) and LX-1 (**b**) tumor bearing mice. Namawala (human lymphoma model) or LX-1 (human SCLC model) tumor cells were implanted subcutaneously into SCID mice. Mice received vehicle (*dotted line*), VCR (*dashed lines*), or VSLI (*solid lines*) on days 11, 18 and 25 post-implantation. Data represent mean ± SD, *N* = 5–10. The symbols indicate the following treatments: Vehicle-treated control (−), vincristine 0.5 mg/kg (+), 1.0 mg/kg (*open circle*), 1.5 mg/kg (*open square*); VSLI 1.0 mg/kg (*filled circle*), 1.5 mg/kg (*filled square*), 2.5 mg/kg (*filled diamond*)

The prolonged plasma circulation time and increased VCR penetration and concentration in tissues (i.e., passive targeting) of VSLI translated into enhanced antileukemia activity compared to standard VCR without additional toxicity (i.e., widens the therapeutic index). Examples of this are shown in Fig. 4. Mice bearing Namwala xenograft tumors treated with VSLI showed better tumor growth suppression at 1.0, 1.5 and 2.5 mg/kg compared to animals treated with 0.5, 1.0 or 1.5 mg/kg VCR (Fig. 4a). Tumor growth suppression was dose dependent for both agents; the maximum tolerated dose for VSLI was 2.5 mg/kg versus 1.5 mg/kg for VCR. The antitumor activity of VSLI was greater at each dose level with maximal activity at the MTD of 2.5 mg/kg, a dose unachievable with standard formulation VCR. Similarly, in the LX-1 human small-cell lung carcinoma model, VSLI demonstrated greater antitumor activity than an equivalent dose of VCR at 1.0 mg/kg administered q7d × 3 (Fig. 4b) [48].

The superior antitumor activity of VSLI compared to that of VCR was further demonstrated in a variety of human tumor xenograft and murine syngeneic models representing several cancer types. Table 4 provides a summary of studies conducted using either a single administration or a multi-dose regimen. Overall, in 18 animal tumor models representing 11 different cancer indications, VSLI was more active than VCR in 11 tumor models representing 8 cancer indications (Table 4). Improved activity of VSLI over VCR was observed using a variety of dosing schedules and routes of tumor implantation. In all cases, VSLI antitumor activity was equivalent to or exceeded that of the same dose level of VCR; in no case was VSLI activity less than that seen at an equivalent dose level of VCR. The antitumor activity of VSLI was dose dependent in 13/18 of the tumor models evaluated (Table 4). Three models were not sensitive to either VSLI or VCR (B16/BL6 melanoma, NCI-H460 NSCLC, and HT29 colon carcinoma) while one (SR lymphoma) was very sensitive to both VSLI and VCR. Further, as measured by changes in body weight or mortality, VSLI was tolerated at higher dose levels than VCR. Combined, these nonclinical data demonstrate that VSLI has potent antitumor activity which exceeds that of conventional VCR.

**Table 4:** Summary of VSLI versus VCR antitumor activity in 18 human tumor xenograft models

Tissue origin	Tumor model^a^	Schedule^b^	Sensitivity to VCR	Dose-dependent antitumor activity	Relative antitumor activity
Leukemia	P388^c^ (IP)	Single	Moderate	VSLI	VSLI > VCR*
Lymphoma	Namalwa	q7d × 2, q7d × 3	Moderate	VSLI, VCR	VSLI > VCR*
Lymphoma	RL	q7d × 3	High	VSLI, VCR	VSLI > VCR
Lymphoma	DoHH2	q7d × 3	High	VSLI, VCR	VSLI = VCR
Lymphoma	SR	q7d × 2	High	None	Unable to assess
SCLC	LX-1	Single	Weak	VSLI	VSLI > VCR
SCLC	LX-1	q7d × 3	Moderate	NA	VSLI > VCR*
SCLC	NCI-H69	Single	High	VSLI, VCR	VSLI = VCR
SCLC	DMS273	Single	Moderate	VSLI, VCR	VSLI = VCR
Breast carcinoma	MX-1	Single	Moderate	VSLI, VCR	VSLI > VCR
Breast carcinoma	MX-1	q21d × 2, q21d × 3	High	VSLI, VCR	VSLI > VCR*
Breast carcinoma	MX-1	q7d × 3	High	VSLI, VCR	VSLI > VCR
Breast carcinoma	MX-1	q7d × 3	High	VSLI, VCR	VSLI > VCR
Renal carcinoma	RXF393	Single	Weak	VSLI	VSLI > VCR*
Prostate carcinoma	PC-3	Single	Moderate	VSLI	VSLI > VCR
Prostate carcinoma	PC-3	q7d × 3	High	VSLI, VCR	VSLI = VCR
Prostate carcinoma	PC-3 (OT)	q7d × 3	Moderate	None	VSLI ≥ VCR
Kaposi’s sarcoma	My1	q7d × 3	Weak	VSLI, VCR	VSLI ≥ VCR
Melanoma	B16/BL6^c ^(IV)	Single	Not sensitive	None	Unable to assess
Melanoma	B16/BL6^c^	q7d × 3	Not sensitive	None	Unable to assess
NSCLC	NCI-H460	Single	Not sensitive	None	Unable to assess
Colon carcinoma	HT29	Single	Not sensitive	None	Unable to assess
Multiple myeloma	LAGκ-1A	q7d × 3	Moderate	VSLI	VSLI > VCR
Multiple myeloma	LAGκ-1B	q7d × 3	Not sensitive	VSLI	VSLI > VCR

*IV* intravenous, *IP* intraperitoneal, *NA* not applicable (only one dose tested), *OT* orthotopic, *SCLC* small-cell lung cancer

^a^Tumors were human xenograft models implanted subcutaneously, unless specified

^b^Repeat-dose schedules were once a week for 2 or 3 cycles (q7d × 2 or q7d × 3) or every 3 weeks for 2 or 3 cycles (q21d × 2 or q21d × 3). ^c^Murine tumor model

* Statistically significant difference (*p* < 0.05)

Data from Leonetti et al. [49] demonstrated that VSLI is also effective in three drug-resistant tumor models that over-express *P*-glycoprotein. When treated with standard VCR, M14 melanoma, MCF-7 breast carcinoma and LoVo colon carcinoma cells showed growth delay, whereas drug-resistant variants of each of those cell lines were resistant to growth inhibition by standard VCR. Xenograft tumors grown in mice from each of those cell lines were sensitive to standard VCR, whereas their resistant variants showed no delay in tumor growth. In contrast, tumors from both the parental cell lines and the drug-resistant variants were sensitive to VSLI and resulted in significant tumor growth delay. Immunohistochemical analysis of VSLI-treated tumors further demonstrated massive necrosis and apoptosis. These data suggest that VSLI may be effective in drug-resistant tumors that express increased levels of *P*-glycoprotein.

## VSLI clinical experience

**Fig. 5:**
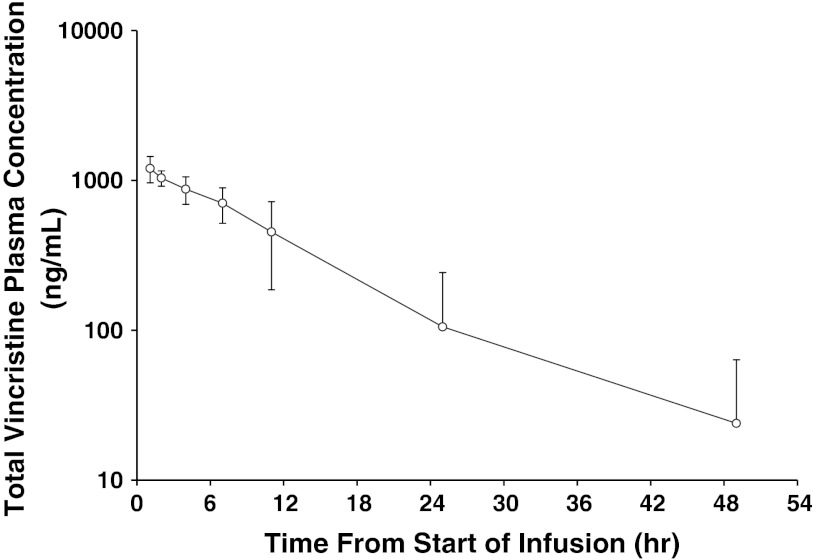
Mean plasma concentration–time profile of total VCR in humans following IV administration of VSLI at 2.25 mg/m^2^. Plasma was collected from adult Ph-chromosome-negative relapsed/refractory patients, *N* = 12. Total VCR concentrations were measured at the indicated times post-dose of VSLI using a validated LC/MS–MS method. The lower limit of quantitation (LLOQ) for vincristine sulfate was 1.00 ng/mL. Pharmacokinetic parameters for total VCR concentrations (encapsulated and free) in plasma were calculated from the plasma concentration–time data using a noncompartmental analysis (NCA) method (WinNonlin Professional Network Edition, Version 5.2, Pharsight Corp, Palo Alto, CA, USA)

The pharmacokinetics of VSLI in humans is similar to that observed in nonclinical species. Specifically, VSLI is a long circulating, slow-release nanoparticle formulation of VCR that remains within the plasma compartment for a prolonged period of time compared to standard VCR (Fig. 5; Table 4). Due to the slow release of VCR from the liposome, the plasma concentration profile of total VCR represents that of the encapsulated drug. Unlike the very rapid distribution phase observed with standard VCR, a delay of 3–12 h in VCR clearance from plasma is observed following VSLI administration, resulting in total VCR levels that remain relatively constant before declining with time. This delay phase contributes significantly to the plasma AUC following VSLI administration. Subsequent to this delay phase, a wide variance in profiles is observed, ranging from apparent monoexponential declines in plasma concentrations of total VCR to a range of apparent bi-exponential profiles. This variability in pharmacokinetic profile represents the clearance of the liposomes from the plasma and the capacity of the MPS system to mediate that clearance. Importantly, no differences in tolerability were seen between subjects with monoexponential and bi-exponential profiles. And gender, age, BSA, or cancer type did not affect Cl or exposure (AUC_inf_) (Table 5).

**Table 5:** Mean plasma PK parameters of total vincristine in humans following IV administration of VSLI at 2.25 mg/m^2^

	*C*_max_ (ng/mL)	*T*_max_ (h)	AUC_0–last_ (ng h/mL)	AUC_0-inf_ (ng h/mL)	*t*_1/2_ (h)	CL (mL/h)	Vd (mL)	Vd_ss_ (mL)	MRT_0–inf_ (h)
*N*	13	13	13	12	12	12	12	12	12
Mean	1,220	–	13,732	14,566	7.66	345	3,569	2,914	9.63
SD	229	–	5,666	6,368	3.18	177	1,924	1,219	4.44
CV %	18.8	–	41.3	43.7	41.5	51.2	53.9	41.8	46.1
Min	919	1.08	6,975	7,036	4.49	148	1,540	1,803	5.38
Median	1,230	1.25	13,502	13,680	6.61	302	3,254	2,601	8.43
Max	1,720	4.17	24,036	26,074	12.6	783	7,754	6,500	17.7

VSLI has been studied in 20 clinical trials and 2 compassionate use programs. Malignancies represented in these trials include acute lymphoblastic leukemia (ALL), non-Hodgkin’s lymphoma (NHL), Hodgkin’s lymphoma, and solid tumors such as metastatic melanoma and lung cancer. Both adults and children have been studied. As a result of these studies and the strong historical data demonstrating clinical activity of the standard VCR in hematologic cancers, VSLI is being developed in ALL and other hematologic malignancies.

ALL is a malignant disease of B- or T-lymphocytes characterized primarily by failure of proper cellular maturation and consequent aberrant cell proliferation. Malignant proliferation of lymphoblasts in the bone marrow and blood suppresses normal hematopoesis and may lead to infiltration of extramedullary sites such as the liver, spleen, thymus, meninges and gondads [50]. The disease is characterized by rapid progression and death if not successfully treated. Sequential modifications of treatment protocols have led to remarkable improvement in survival outcome in pediatric and adolescent ALL patients with survival rates exceeding 80 %. Despite the remarkable success in treatment of childhood and adolescent ALL, adult ALL patients are underserved by existing treatment options as reflected by the poor survival rates and extremely poor outcomes in the relapsed setting. Recent trials have shown that 65–85 % of adults will achieve a complete remission; however, the duration of these responses is often short, especially in older adults [51, 52]. When relapsed, adult ALL has a poor long-term survival rate of 20–40 % [53, 54]. Currently there is no clear standard of care or guidance for the treatment of advanced, relapsed and/or refractory ALL.

Based on the clear unmet medical need, the superiority of VSLI over standard VCR in nonclinical studies and encouraging activity in Phase 1 trials, a multi-national pivotal, Phase 2, single-arm, open-label trial (NCT00495079) of high dose (2.25 mg/m^2^), weekly VSLI monotherapy was conducted in heavily pre-treated adults with advanced, relapsed and refractory B or T cell lineage Philadelphia chromosome-negative (Ph-) ALL. VSLI monotherapy resulted in meaningful clinical outcomes; the CR/CRi rate and overall response rates were 20 % and 35 %, respectively [55]. VSLI monotherapy was effective as third-, fourth-, and fifth-line therapy and in patients refractory to other single- and multi-agent therapies. The median uncensored CR/CRi duration was 23 weeks (range 5–66 weeks), and 5 patients were long-term survivors. Importantly, 12 patients who were ineligible for immediate hematopoietic cell transplant were able to subsequently receive a transplant. The toxicity profile of high-dose VSLI was predictable, manageable and comparable to that of standard dose and formulation VCR despite the delivery of large, normally unachievable, individual and cumulative doses of VCR. These studies led to the accelerated approval of VSLI by the FDA for the treatment of adult patients with Ph- ALL in second or greater relapse or whose disease has progressed following two or more antileukemia therapies.

## Summary

VSLI is a proprietary sphingomyelin- and cholesterol-based nanoparticle formulation of VCR that was designed to be different from and overcome the dosing and pharmacokinetic limitations of standard VCR. In nonclinical studies, VSLI: 1) increases the plasma circulation time; 2) increases tumor tissue delivery by preferential extravasation from fenestrated (“leaky”) vasculature; 3) accumulates in tumor tissues; and 4) slowly releases VCR in tumor tissues instead of the systemic circulation. These unique pharmaceutical properties resulted in superior nonclinical pharmacokinetic properties in mice, rats and dogs and translated into increased efficacy in 11 murine tumor models compared to standard VCR. Clinical trials demonstrated safety, tolerability, and promising activity of VSLI in adults with advanced relapsed/refractory leukemia and lymphoma. VSLI recently received accelerated FDA approval.
